# Localized Spoof Surface Plasmons based on Closed Subwavelength High Contrast Gratings: Concept and Microwave-Regime Realizations

**DOI:** 10.1038/srep27158

**Published:** 2016-06-02

**Authors:** Zhuo Li, Bingzheng Xu, Liangliang Liu, Jia Xu, Chen Chen, Changqing Gu, Yongjin Zhou

**Affiliations:** 1 Key Laboratory of Radar Imaging and Microwave Photonics, Ministry of Education, College of Electronic and Information Engineering, Nanjing University of Aeronautics and Astronautics, Nanjing, 211106, China; 2 State Key Laboratory of Millimeter Waves, Southeast University, Nanjing, 210096, China; 3 Department of Physics, College of Liberal Arts and Sciences, Arizona State University, 871504, USA; 4 Department of Electrical and Computer Engineering, National University of Singapore, 4 Engineering Drive 3, 117576, Singapore; 5 Key Laboratory of Specialty Fiber Optics and Optical Access Networks, Shanghai University, Shanghai, 200072, China

## Abstract

In this work, we report the existence of spoof localized surface plasmons (spoof-LSPs) arising with closed high contrast gratings (HCGs) at deep subwavelength scales, another platform for field localization at microwave frequencies. The HCGs are in the form of a periodic array of radial dielectric blocks with high permittivity around a metal core supporting spoof-LSPs of transverse magnetic (TM) form. Simulation results validate the phenomenon and a metamaterial approach is also given to capture all the resonant features of this kind of spoof-LSPs. In addition, experimental verification of the existence of spoof-LSPs supported by a three dimensional (3D) HCGs resonance structure in the microwave regime is presented. This work expands the original spoof-LSPs theory and opens up a new avenue for obtaining resonance devices in the microwave frequencies.

Surface plasmons (SPs) are coherent delocalized electron oscillations that exist at the interface between a dielectric and a conductor at optical frequencies, which have been a hot spot due to the features of field enhancement and subwavelength field confinement^1^,^2^,^3^. Generally, SPs can be classified into two types: surface plasmon polaritons (SPPs)^4^ which are propagating modes that travel along a metal-dielectric interface and localized surface plasmons (LSPs)^5^ which are resonance modes supported by subwavelength metal particles. The properties of natural LSPs have long been of interest in increasing applications in optical antenna, near field optics and surface enhanced spectroscopy^5^,^6^,^7^. Almost all the studies concerning SPPs and LSPs have been confined to ultraviolet, visible and near infrared light regime until 2004 when Pendry *et al*. proposed an effective approach by patterning metal surface with periodical grooves or holes to significantly improve the confinement of SPPs in the perfect electric conductor (PEC) limit, which is the origin of spoof SPPs developed at lower frequencies^8^,^9^,^10^,^11^,^12^,^13^,^14^,^15^,^16^,^17^. In 2012, Garcia-Vidal *et al*. demonstrated that periodically textured closed surface can support LSPs also in the PEC limit, namely spoof-LSPs^18^. Since then, the existence of spoof-LSPs has been verified through experiments in various kinds of models. Multipolar spoof-LSPs on a planar textured metallic disk are proposed and experimentally demonstrated at microwave frequencies^19^. The existence of magnetic LSPs that are supported by cylindrical metal structures corrugated by very long, curved grooves has been theoretically proved and experimentally verified^20^. More recently, multi-band spoof-LSPs have been realized by decorating periodically textured cylinder with multiple groove depths^21^. Later on, the existence of spoof-LSPs in periodically textured PEC circular cavities with the same or multiple different grooves has been reported^22^,^23^. In these works, the textured metal surfaces can be regarded as subwavelength metallic gratings, which can be modeled as an anisotropic and homogeneous medium layer around or in a metal core, resembling nearly the same characteristics of the LSPs in the optical regime. Dielectric gratings, especially high contrast gratings which have many distinct attributes including broadband ultra-high reflectivity and transmission, very high-Q factor resonance, have been employed in many optoelectronic devices^24^,^25^,^26^,^27^. More recently, Li^28^ theoretically demonstrated that subwavelength HCGs on a PEC plane can also support spoof surface plasmons, which is a generalization of spoof surface plasmons originated from subwavelength metallic gratings. Thus, it is also of great interest and value to study whether spoof-LSPs could also arise with closed subwavelength high contrast gratings around a metal core, which is the motivation of this paper.

In this work, we expand the concept of spoof-LSPs and demonstrate that closed subwavelength HCGs around a metal core can support spoof-LSPs both in two dimensional (2D) and 3D cases. With theoretical analysis and simulations, we show that HCGs in the form of a periodic array of radial dielectric blocks with high permittivity can support spoof-LSPs of transverse magnetic (TM) form, which is further validated through a metamaterial approximation to capture all the resonant features. Additionally, simulations and experimental verification of spoof-LSPs supported by a 3D HCGs structure is presented to validate our theory. This work is a natural extension of the original concept of spoof-LSPs and can find potential applications in tunable designer-plasmonic sensors.

## Results

### Spoof LSPs on a 2D closed subwavelength HCGs resonance structure

**Figure 1:**
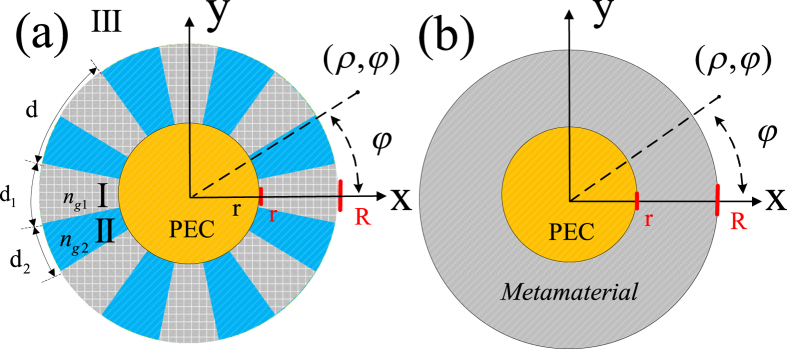
(**a**) A 2D HCGs layer around a cylindrical PEC core with the inner and outer radii *r* and *R*, periodicity *d*, and height *h* = *R* − *r*. The refraction indices of radial Blocks I and II are *n*_*g*1_ and *n*_*g*2_ and the widths are *d*_1_ and *d*_2_. (**b**) An effective medium approximation of the geometry displayed in (**a**) is an anisotropic and homogeneous layer of thickness *h* = (*R* − *r*) wrapped around a PEC cylinder of radius *r*.

Now we focus on the 2D closed textured surface in Fig. 1(a), which consists of a HCGs layer around a PEC cylindrical core. The HCGs layer stretching from the inner radius *r* to the outer radius *R* is a periodic dielectric array of two radial Blocks I and II with refractive indices *n*_*g*1_ and *n*_*g*2_ in one periodicity *d* = 2*πR*/*N*, where *N* is half the total number of radial blocks. Block I and II are of the same height *h* = *R* − *r* and different width *d*_1_ and *d*_2_. Subwavelength condition can always be satisfied by adjusting the number of the blocks and the radius *R* to obtain *d* ≪ *λ*_0_ (*λ*_0_ is the wavelength of the incident wave in free space). The outer radius *R* is set to the unit length considering that the resonance frequencies scale with the reciprocal size of the structure. In this work, we set Block II as air with *n*_*g*2_ = 1(*ε*_*r*2_ = *μ*_*r*2_ = 1) and Block I as dielectric medium with high permittivity *ε*_*r*1_ = 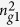 (*μ*_*r*1_ = 1).

**Figure 2:**
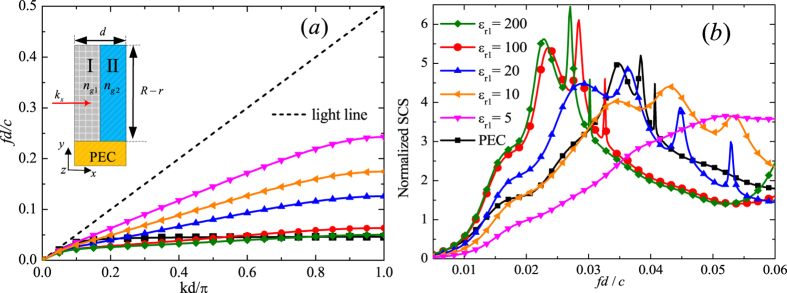
(**a**) Variation of the dispersion curves of the spoof surface plasmons that are supported by HCGs above a PEC plane with different *ε*_*r*1_ compared with the PEC case. (**b**) The calculated normalized scattering cross sections of the 2D textured structure with different *ε*_*r*1_ compared with the PEC case.

First, we consider a specific case when *N* = 60, *d*_1_ = *d*_2_ = *d*/2 and *r* = *R*/3 and calculate the revolution of dispersion curves of the corresponding spoof SPPs that would propagate on a flat HCGs in the same size above a PEC plane with the variations of *ε*_*r*1_ from 5 to 200 shown in Fig. 2(a). Although the trend of these dispersion curves are quite different from those of textured PEC surfaces in the same size, they are all on the right-hand side of the light line and have asymptotic frequencies, at which the group velocity becomes zero. All these characteristics can also be found in spoof SPPs that are supported on the textured PEC surfaces^8^. The normalized scattering cross sections (SCSs) spectrums of the same structure with the increase of *ε*_*r*1_ from 5 to 200 are shown in Fig. 2(b), from which we can clearly observe the red shift of all the resonance peaks with increasingly narrower bandwidth. The SCSs spectrum for the same structure of PEC material is also given for comparison. We remark the normalized SCSs are the real SCSs divided by the diameter 2*R*. Nowadays, dielectrics with high permittivity constant *ε*_*r*1_ up to 100 with ultra-low losses (typically loss angle tangent less than 0.0001) at microwave frequencies can be easily fabricated using sintered MgCO_3_-based composites. In the following simulations, we set *ε*_*r*1_ = 100 without any loss for simplicity at microwave frequencies.

**Figure 3:**
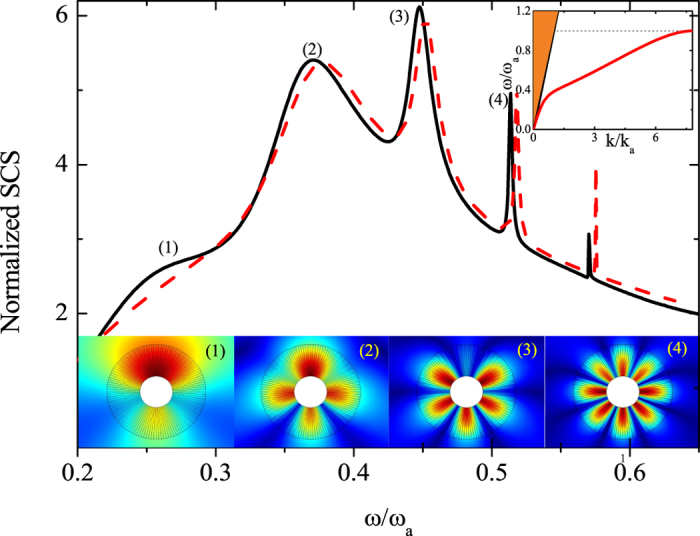
The calculated normalized SCSs of the model in Fig. 1(a) (black solid line) and of the metamaterial approximation in Fig. 1(b) (red dashed line) with *r* = *R*/3, *N* = 60, *d*_1_ = *d*_2_, *ε*_*r*1_ = 100 and *ε*_*r*2_ = 1. The inset shows the dispersion relation of the corresponding spoof SPPs that would propagate on a flat PEC-based HCGs in the same size. The absolute |**H**| field at the marked peaks 1–4 corresponding to the dipo-, quadru-, hexa-, and octopole modes are given in the bottom panels under TM-polarized plane wave excitations.

For probing the plasmonic resonances, a TM-polarized incident plane wave (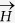 pointing along the *z* direction) propagating along the −*y* axis is considered. In Fig. 3, the normalized SCSs are presented in black solid line as a function of the normalized frequency for a representative HCGs around a PEC core (*r* = *R*/3, *N* = 60, *d*_1_ = *d*_2_ = 0.5*d, ε*_*r*1_ = 100) calculated using the commercial software COMSOL MULTIPHYSICS. We remark that the frequency is normalized to the asymptotic frequency *ω*_*a*_ of the corresponding spoof SPPs that would propagate on a PEC-based flat HCGs. The dispersion relation of these spoof SPPs and the free-space light dispersion in normalized units (*k*_*a*_ = *ω*_*a*_/*c*) are shown in the inset of Fig. 3. The asymptotic frequency is mainly controlled by the dielectric block height *h* and medium parameters of the HCGs layer. It can be observed that there are multiple peaks in the SCSs spectrum which are closely related to the resonant EM behavior of the HCGs cylinder. In Fig. 3, the marked peaks 1–4 are directly related to the excited spoof-LSPs resonances and the bottom panels display the magnetic field amplitude 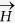 at the di-, quadru-, hexa- and octopole resonances, whose distributions are concentrated both in Blocks I and II within the HCGs layer and are quite different from that of ref. 18, in which most of the magnetic fields exist in the metal grooves. We can find that resonances of dipo- and quadrupole modes are not very strong, exhibiting manifest asymmetric field distributions due to the asymmetry of excitations. The incident field would not feel the details of the HCGs structure for the periodicity of the HCGs is in the subwavelength scale. Thus we can uncover the physics behind this spectrum by applying the concept of an EM metamaterial that the HCGs layer can be interpreted as an equivalent medium of thickness *h* = *R* − *r* as illustrated in Fig. 1(b). The effective parameters of the medium in cylindrical coordinates can be defined as^29^:


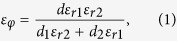



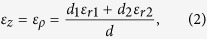


where *ε*_*ρ*_, *ε*_*φ*_ and *ε*_*z*_ are the relative permittivity component in the *ρ, φ* and *z* directions respectively. The relative permeability *μ* of the equivalent medium can be assumed to be isotropic and equals 1. Consequently, in the metamaterial approximation the HCGs layer behaves as an anisotropic and homogeneous layer with specific medium parameters as stated above, which can be put back into the model in Fig. 3 to calculate the normalized SCSs shown in Fig. 3 (red dashed line) with COMSOL MULTIPHYSICS. It is clear that the metamaterial approximation captures all the resonant features of the spectrum, which agrees with the original problem quite well.

### Experimental verification of spoof-LSPs with 3D HCGs structure

**Figure 4:**
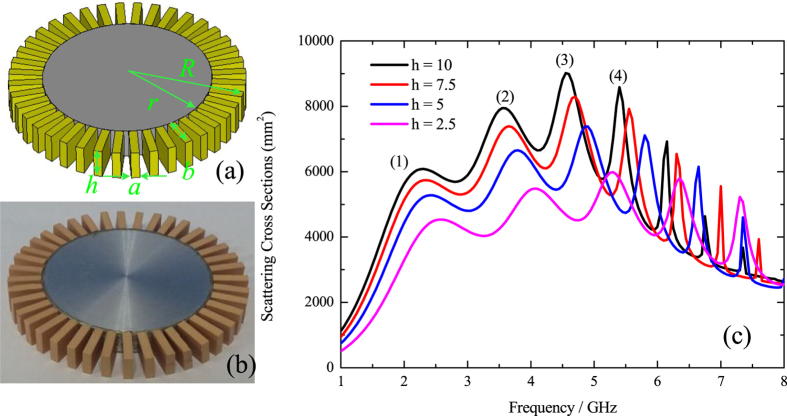
(**a**) The 3D model of HCGs around a PEC cylindrical core. (**b**) The fabricated HCGs around an aluminum cylindrical core. **(c)** The calculated SCSs spectra of the 3D HCGs with *ε*_*r*1_ = 20, *μ*_*r*1_ = 1 around a PEC cylindrical core with different heights (*h* = 2.5 mm, 5 mm, 7.5 mm and 10 mm). Peaks (1)–(4) indicate multipolar plasmonic resonances at dipo-, quadru-, hexa-, and octopole modes, respectively.

**Figure 5:**
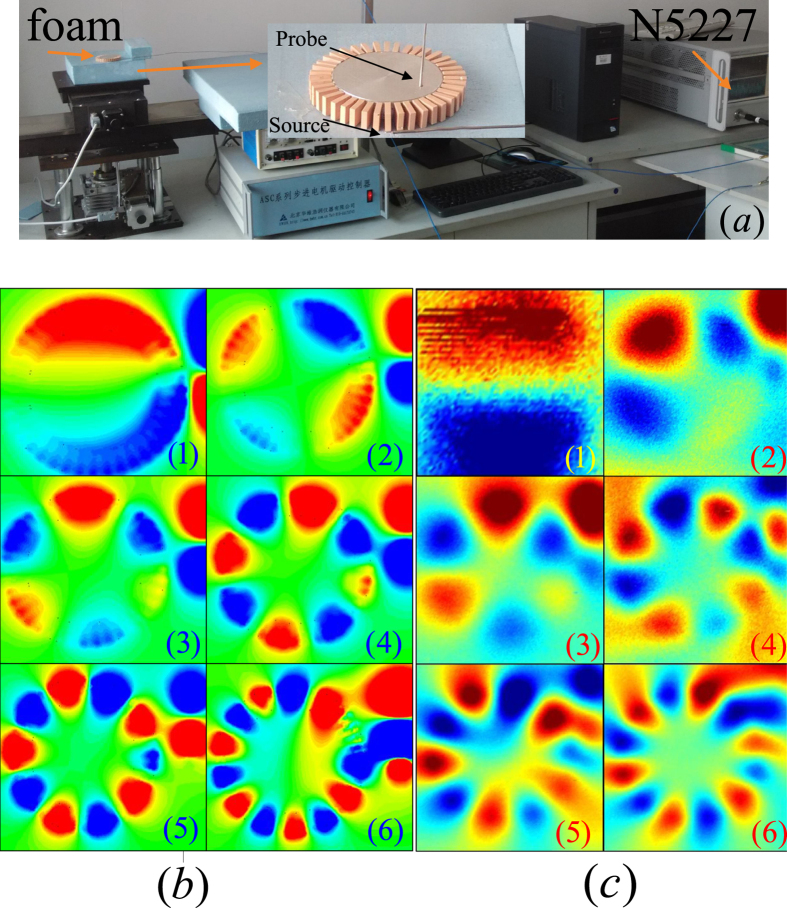
(**a**) Experimental setup for the measurement of the near-field resonances of the HCGs structure. (**b**) The simulated near-field patterns of vertical electric fields *E*_*z*_ on a plane 1.5 mm above the HCGs structure for the dipole mode (M_1_) at 2.3 GHz, quadrupole mode (M_2_) at 3.6 GHz, hexapole mode (M_3_) at 4.64 GHz, octopole mode (M_4_) at 5.54 GHz, decapole mode (M_5_) at 6.3 GHz and dodecapole mode (M_6_) at 6.98 GHz, respectively. (**c**) The measured near-field patterns of vertical electric fields *E*_*z*_ on a plane 1.5 mm above the HCGs structure for the dipole mode (M_1_) at 2.5 GHz, quadrupole mode (M_2_) at 3.8 GHz, hexapole mode (M_3_) at 4.6 GHz, octopole mode (M_4_) at 5.5 GHz, decapole mode (M_5_) at 6.4 GHz and dodecapole mode (M_6_) at 7.2 GHz, respectively.

Now, we focus on the issue whether the spoof-LSPs can still arise in 3D cases. A 3D model and a fabricated one are shown in Fig. 4(a,b) with the outer and inner radii of the structure being *R* = 28 mm and *r* = 20 mm. The HCGs layer consists of a circumferential periodic array of 40 equally-spaced high permittivity radial dielectric blocks with *ε*_*r*1_ = 20 and the inner layer is an aluminum cylindrical core, which can be viewed as PEC in the microwave frequency regime. The width and length of the dielectric block are *a* = 2 mm and *b* = 8 mm, respectively. As the height *h* varies from 2.5 mm to 10 mm with a step of 2.5 mm, the SCSs spectra are calculated using the commercial software CST Microwave Studio to feature all the resonance characteristics shown in Fig. 4(c), from which we can find that multipolar resonances arise with peaks (1)–(4) corresponding to di-, quadru-, hexa-, and octopole modes and as *h* increases, redshifts of all the resonance peaks are observed due to the decreasing of the asymptote frequency of the corresponding spoof SPPs that would propagate on a PEC-based flat HCGs of the same height *h*. As presented in ref. 13, the LSP resonances of a 3D textured metallic disk can also be observed under the excitation of a monopole source. Thus, we fabricate a real HCGs unit with the dielectric ceramic made of sintered MgTiO_3_-CaTiO_3_ powder and attach 40 units uniformly to the side wall of an aluminum cylindrical core to form a HCGs resonance structure (height *h* = 7.5 mm) shown in Fig. 5(a). A monopole source is located 12.5 mm away from the structure and is vertical to the axis of the cylinder core, which is mounted on a foam whose relative permittivity can be regarded as 1. A vertical probe is located 1.5 mm above the resonance structure to capture the near electric field distributions *E*_*z*_. The two ports of vector network analyzer (Agilent N5227A) are connected to the monopole and probe respectively. The simulated and measured near-field distributions of *E*_*z*_ on the plane 1.5 mm above the structure at resonance frequencies are demonstrated in Fig. 5(b,c). The resonance peaks of the measured di-, quadru-, hexa-, octo-, deca-, and dodecapole modes are located at 2.5, 3.8, 4.6, 5.5, 6.4 and 7.2 GHz with a little deviation compared to the simulated resonance peaks located at 2.3, 3.6, 4.64, 5.54, 6.3 and 6.98 GHz due to the fabrication tolerance and alignment error. However, the simulated and measured near fields agree with each other quite well, thus validating our theory of spoof LSPs supported by HCGs structures.

## Discussion

In summary, we have theoretically and experimentally demonstrated that closed HCGs at deep subwavelength scales can support spoof-LSPs with the verifications of metamaterial approximations. A 3D closed HCGs model is built and fabricated to validate our theory with comparison of near field distributions at multipolar resonances between simulation and measurement. Moreover, the HCGs-based spoof-LSPs are distinct from metal-grating based ones in dispersion relation, normalized SCSs and field distributions, which is an expansion of F.J. Garcia-Vidal’s theory of spoof-LSPs and can find potential applications in designing new resonance structures at microwave frequencies.

## Methods

The dispersion curves and SCSs of the 2D model are performed on the commercial software COMSOL Multiphysics. The simulated SCSs curves of the 3D textured HCGs are performed by the commercial software CST Microwave Studio. The HCGs layer of the real 3D resonance strucure is farbricated with dielectric ceramic made of MgTiO_3_-CaTiO_3_ with high dielectric permittivity (*ε*_*r*_ = 20) and low loss angle tangent of less than 0.0001. The experiment was conducted on a near field scanning system developed in the Key Laboratory of Specialty Fiber Optics and Optical Access Networks, Shanghai University.

## Additional Information

**How to cite this article**: Li, Z. *et al*. Localized Spoof Surface Plasmons based on Closed Subwavelength High Contrast Gratings: Concept and Microwave-Regime Realizations. *Sci. Rep.* **6**, 27158; doi: 10.1038/srep27158 (2016).
